# Contained gastric perforation leading to a rare abdominal subcutaneous collection: a case report

**DOI:** 10.1093/jscr/rjaf272

**Published:** 2025-05-15

**Authors:** Sahar Alshammery, Ahmed Alotaibi, Eyad Alwhoaibi

**Affiliations:** General Surgery Department, King Abdullah Bin Abdulaziz University Hospital, Airport Road, King Khalid International Airport, Riyadh 13412, Saudi Arabia; General Surgery Department, King Abdullah Bin Abdulaziz University Hospital, Airport Road, King Khalid International Airport, Riyadh 13412, Saudi Arabia; General Surgery Department, King Abdullah Bin Abdulaziz University Hospital, Airport Road, King Khalid International Airport, Riyadh 13412, Saudi Arabia

**Keywords:** gastric perforation, subcutaneous abscess, peptic ulcer disease

## Abstract

Contained gastric perforations are rare clinical entities, and their extension into the abdominal wall with subcutaneous collection is an exceptionally uncommon presentation. This report highlights the case of a 56-year-old male with a history of uncontrolled diabetes mellitus, chronic non-steroidal anti-inflammatory drugs use, and smoking, presenting with abdominal pain and swelling. Imaging revealed a multiloculated fluid collection extending from a perforated gastric ulcer into the anterior abdominal wall, forming a subcutaneous abscess. The patient was managed conservatively with antibiotics, antifungals, bedside incision and drainage, and proton pump inhibitors. This case underscores the importance of early recognition of atypical presentations of gastric perforation and the role of multidisciplinary management in achieving favorable outcomes.

## Introduction

Gastric perforations, typically resulting from peptic ulcer disease (PUD), are medical emergencies that frequently present with acute peritonitis due to free perforation into the peritoneal cavity. However, in rare cases, the perforation is contained by adjacent structures such as the omentum, leading to localized fluid collections rather than diffuse peritonitis. Even more unusual is the extension of such collections into the anterior abdominal wall, forming subcutaneous abscesses.

Subcutaneous abscesses secondary to gastric perforation present unique diagnostic challenges due to their rarity and often subtle clinical signs. The presence of risk factors such as chronic non-steroidal anti-inflammatory drugs (NSAID) use, uncontrolled diabetes, and smoking further compounds the susceptibility to ulcer formation and complications. This report describes a rare case of a contained gastric perforation with an abdominal subcutaneous collection, highlighting its clinical course, management, and the importance of considering such unusual presentations in patients with risk factors for PUD.

## Case presentation

A 56-year-old male with a history of type 2 diabetes mellitus (HbA1c: 12.8%), PUD, chronic NSAID use, and smoking presented to the emergency department with a 1-week history of abdominal pain and swelling. The pain was sudden in onset, localized to the epigastric region, and resolved spontaneously over time. This was followed by transient abdominal swelling. The patient denied fever, weight loss, nausea, vomiting, changes in bowel habits, hematemesis, or melena.

Around 3 months prior, he had an upper gastrointestinal (GI) bleed due to chronic NSAID use and was treated conservatively. An upper GI endoscopy revealed esophageal white patches but no active bleeding.

**Figure 1:**
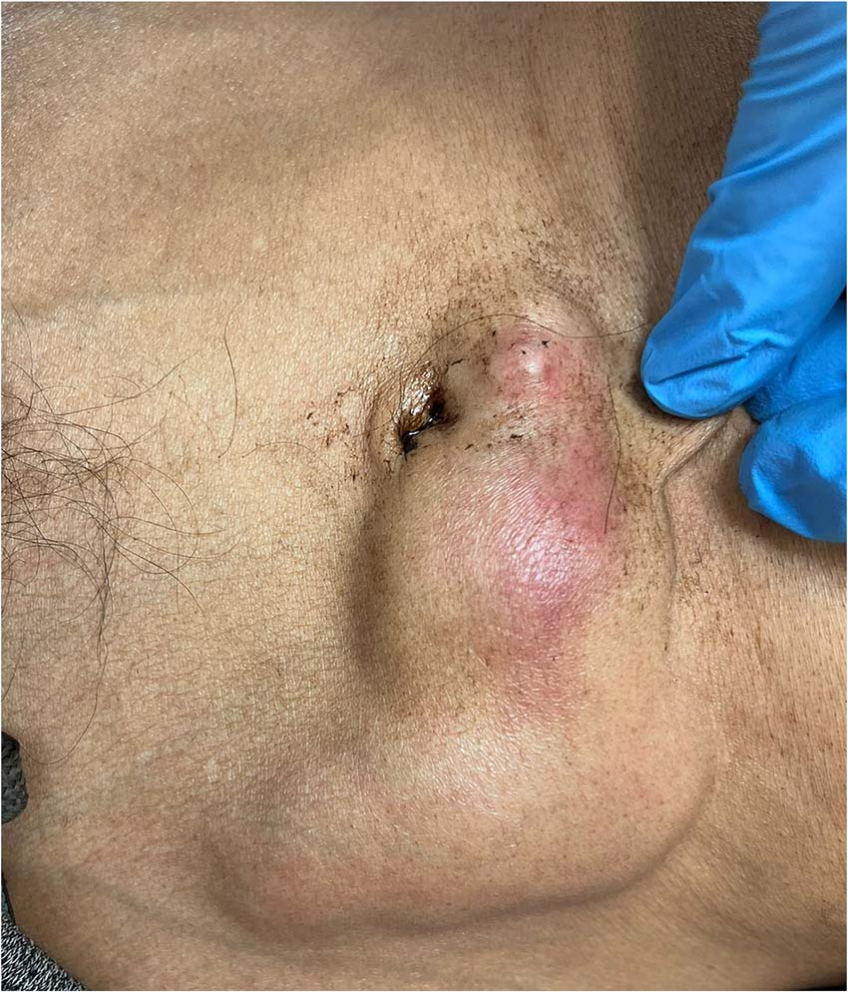
Abdominal examination revealing a non-fluctuant, irregular mass (6 × 7 cm) around the umbilicus, with minimal tenderness and erythema.

On examination, the patient appeared cachectic and exhibited poor hygiene. Abdominal examination revealed a non-fluctuant, irregular mass (6 × 7 cm) around the umbilicus, with minimal tenderness and erythema (Fig. 1).

### Investigations

**Figure 2:**
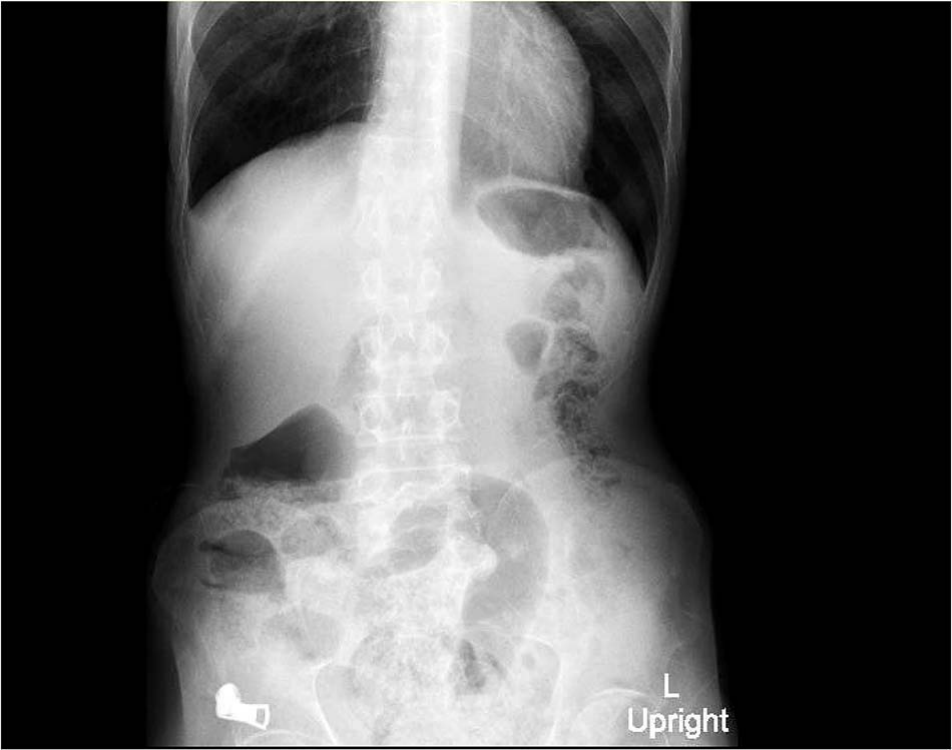
X-ray abdomen (erect) showing non-specific bowel gas distribution; no free air or masses identified.

**Figure 3:**
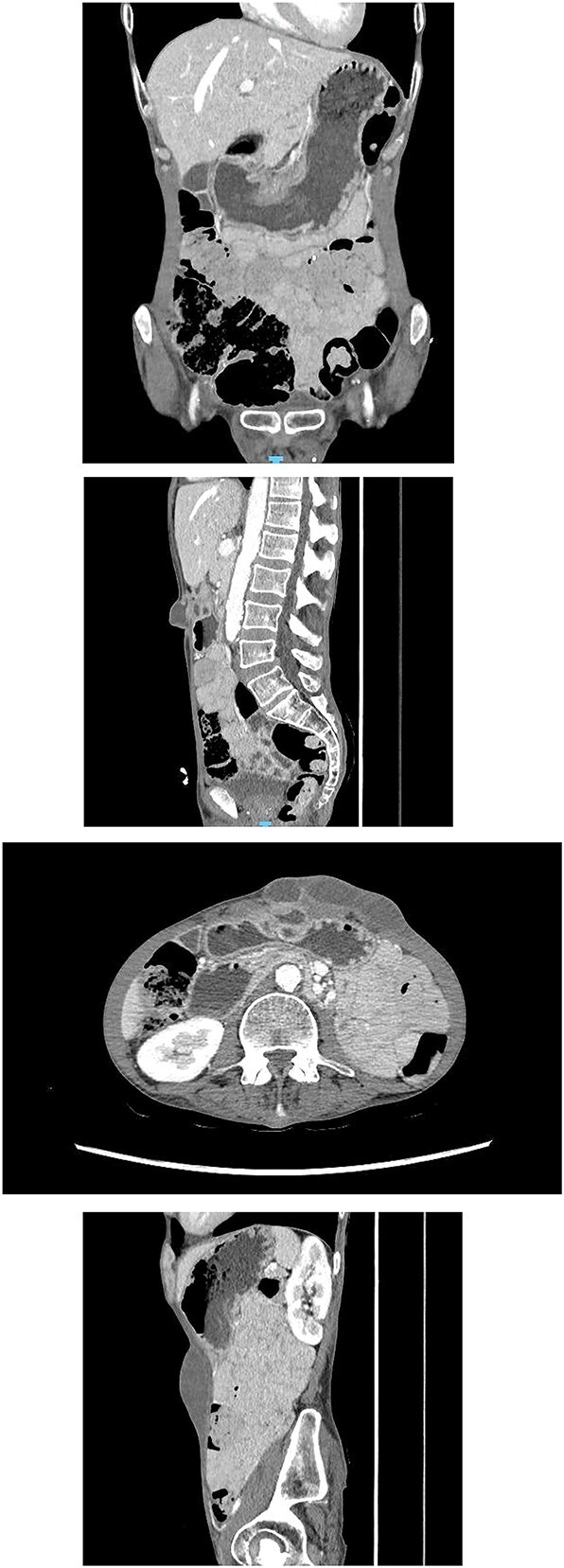
CT abdomen showing a multiloculated fluid collection at the superior greater omentum (7 × 3 × 4.6 cm) inseparable from the gastric antrum, extending into the anterior abdominal wall with a subcutaneous collection (9 × 3.5 × 2.8 cm). No free air or fluid.

**Figure 4:**
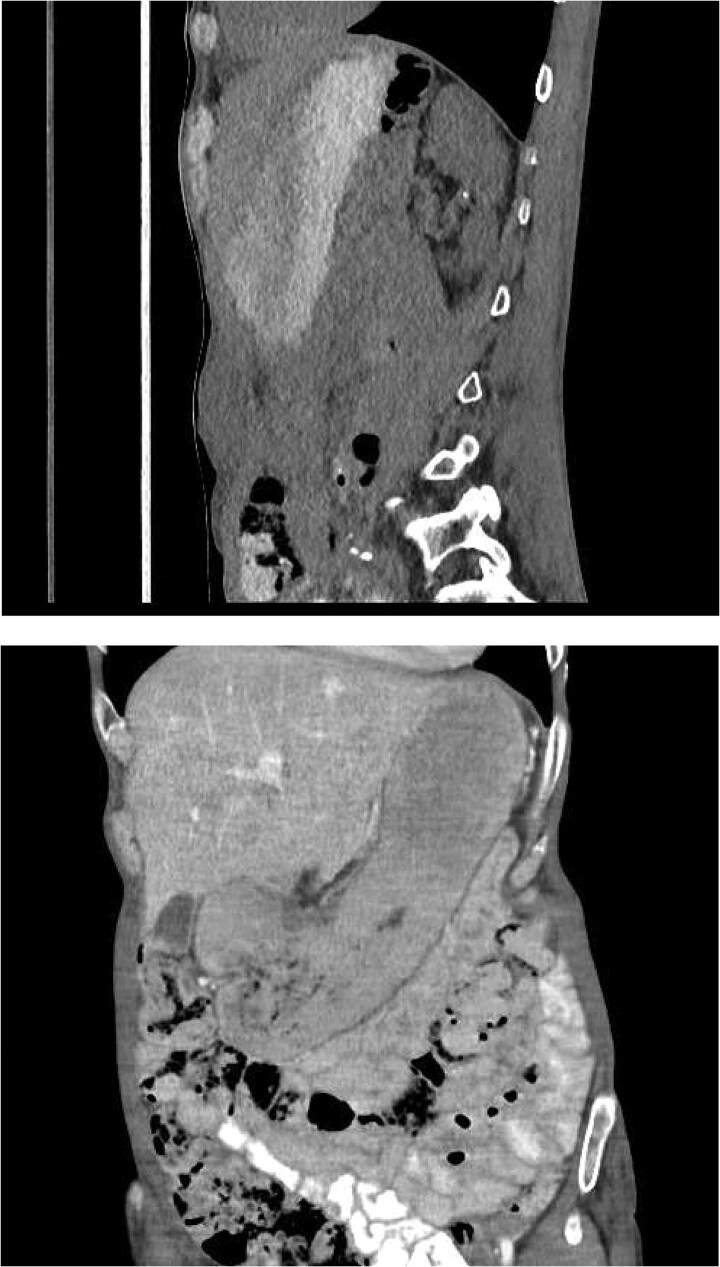
Repeated CT abdomen with oral contrast revealing no oral contrast leak into the upper anterior abdominal fluid collection.

**Figure 5:**
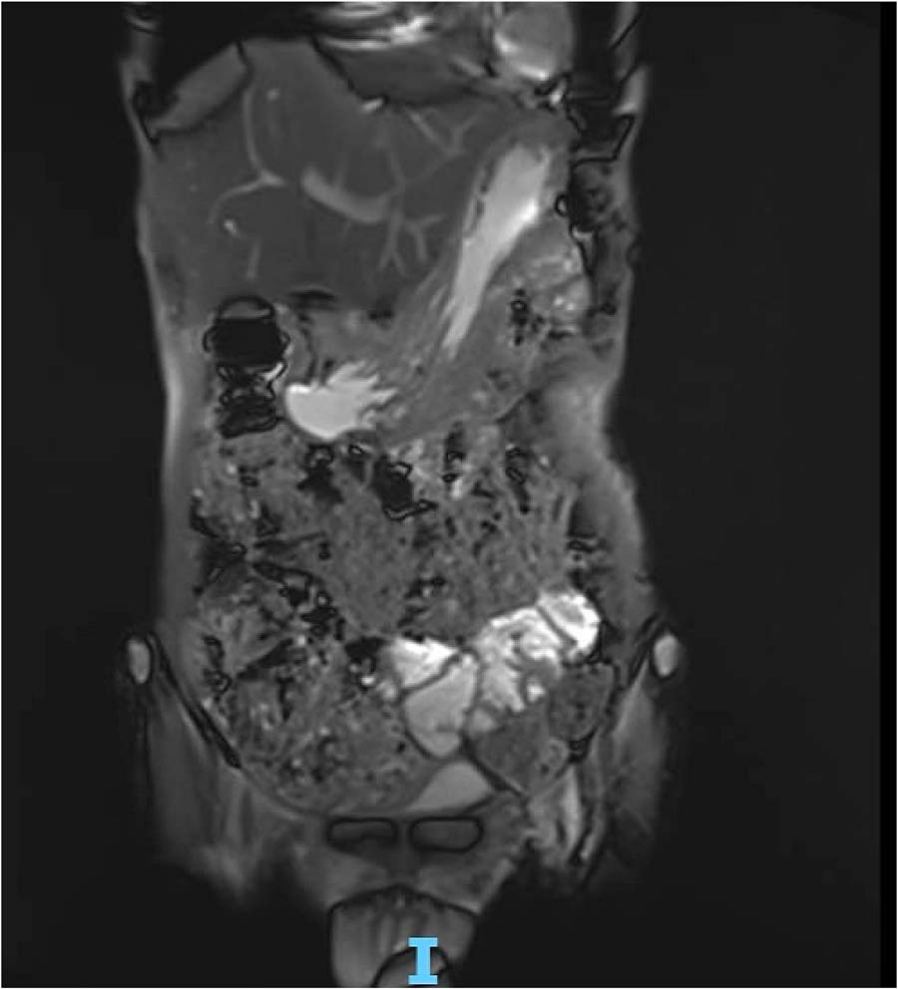
MRE showing gastric wall defect (~0.6 cm) communicating with the collection, consistent with a contained perforation. No evidence of inflammatory bowel disease.

- **Laboratory Results**:
  - Hb: 11.7 g/dL
  - WBC: 8 × 10^9^/L
  - PLT: 250 × 10^9^/L
  - Lipase: 48 U/L
  - Amylase: 60 U/L
  - CRP: 2.9 mg/L
  - HbA1c: 12.8%
- **Imaging**:
  - X-ray abdomen (erect) (Fig. 2): Non-specific bowel gas distribution; no free air or masses identified.
  - CT abdomen (Fig. 3): A multiloculated fluid collection at the superior greater omentum (7 × 3 × 4.6 cm) inseparable from the gastric antrum, extending into the anterior abdominal wall with a subcutaneous collection (9 × 3.5 × 2.8 cm). No free air or fluid.
  - Repeated CT abdomen with oral contrast (Fig. 4): No oral contrast leak into the upper anterior abdominal fluid collection.
  - Magnetic resonance enterography (MRE) (Fig. 5): Gastric wall defect (~0.6 cm) communicating with the collection, consistent with a contained perforation. No evidence of inflammatory bowel disease.

### Management

The patient was admitted under the General Surgery team and managed conservatively due to the inaccessibility of interventional drainage:

- Kept NPO, initiated IV fluids.
- Empiric antibiotics (piperacillin-tazobactam) and antifungals.
- Subcutaneous collection underwent bedside incision and drainage (I&D), with fluid sent for microbiological culture. The culture revealed heavy growth of *Candida albicans*, with no bacterial pathogens isolated.
- Gastroenterology consultation recommended high-dose PPI (IV pantoprazole) and a delayed endoscopy in 6 weeks due to high procedural risk.
- The patient’s clinical status improved with supportive care. He was started on an oral diet, tolerated well, and discharged on oral triple therapy (omeprazole, clarithromycin, amoxicillin) for *Helicobacter pylori*.

### Outcome and follow-up

The patient was scheduled for follow-up appointments with General Surgery and Gastroenterology, but he failed to attend.

## Discussion

Contained gastric perforations with subcutaneous abdominal wall collections are exceedingly rare. In this case, the gastric perforation led to a multiloculated perigastric collection extending into the anterior abdominal wall, forming a subcutaneous abscess. This rare presentation highlights the need for high clinical suspicion and comprehensive imaging in patients with atypical abdominal findings.

Similar cases have been reported in the literature, emphasizing the variability in presentations of peptic ulcer perforations. For example, an anterior abdominal abscess secondary to a duodenal perforation was reported in a 65-year-old female, where imaging played a pivotal role in identifying the connection between the abscess and the gastrointestinal tract [1]. Another report described a perforated gastric ulcer leading to intra-abdominal abscess formation, further illustrating the potential for localized collections in atypical locations [2]. A similar case involving a perforated duodenal ulcer with an anterior abdominal abscess has also been documented, reinforcing the need for timely diagnosis and imaging in complex presentations [3].

The rarity of this presentation underscores the importance of individualized management strategies. In this patient, bedside I&D combined with empiric antifungal and antibacterial therapy led to clinical improvement. While conservative management is appropriate for hemodynamically stable patients, adherence to follow-up is critical to prevent complications or recurrence.
